# Dermoid cyst of the mandibula: a case report

**DOI:** 10.1186/1757-1626-1-260

**Published:** 2008-10-22

**Authors:** Dardo Menditti, Luigi Laino, Nicola Ferrara, Alfonso Baldi

**Affiliations:** 1 Dipartimento di Scienze Odontostomatologiche, Seconda Università di Napoli, Italia; 2 Dipartimento di Biochimica, Sezione di Anatomia Patologica, Seconda Università di Napoli, Italia

## Abstract

**Background:**

Dermoid cysts are rare congenital lesions derived from pluripotential cells.

**Case Presentation:**

We present a case of a female patient aged 30 years presenting for a lesion of the mandibula incidentally seen on a routine panoramic radiograph obtained for dental care. The instrumental, clinical and histological characteristics of the lesion are reported. Finally, a bibliographic revision of this pathology at the level of the oral cavity is reported.

**Conclusion:**

Dermoid cysts must be considered in the differential diagnosis of mandibula's lesions.

## Introduction

Dermoid cysts are rare congenital lesions derived from pluripotential cells. Nowadays their etiology is unknown. Embriologically, the most reliable theory is that trapped pluripotential cells are a result of the inclusion error, during the early weeks of the intrauterine life, and then they develop into one or into all the three ectoderm, mesoderm and endoderm tissues. The differentiation of these germ layers can produce skin adnexa (such hair, sudoriparous and sebaceous glands), muscle, bone, cartilage, teeth, and mucous membranes. The reason of this occurrence could be any trauma, infection or spontaneous autonomous new growth [1,2].

Therefore considering these lesions, three varieties may be classified by their histological aspects: epidermoid cyst is lined by stratified squamous epithelium and it's composed of one of the germinal layers (ectodermic); dermoid cyst is lined by stratified epithelium with skin adnexa; teratoid can be cystic or solid featured. In the current literature the term "dermoid cyst" often refers to all types of these lesions [1,2].

Dermoid cysts are frequently found in sites where embryonic parts fuse together. The majority of reported cases are in the midline of the body, as well as in the ovaries and in the testicles. There is no sex predilection. Their appearance occurs about the second or third decade of life as slowly enlarging masses. The treatment is surgical removal.

In the oral cavity they are also classified as non-odontogenic cystic lesions (accounting for 1,6% of the total); the most frequent sites are the midline of the floor of the mouth (sublingual or submental) and hard palate [3]. Rare cases are reported in the tongue, in the cheek, in the parotid gland and very rare cases in the maxilla and mandibula [4]. They are asymptomatic but their slow enlargement can cause obstruction with consequent dysphagia, dysphonia, and at last dyspnea. The size of dermioid cysts is very variable (up to ten cm in diameter) and it depends on their first clinic manifestation [5].

We report on a very rare case of epidermoid cyst of the mandibula.

## Case presentation

**Figure 1:**
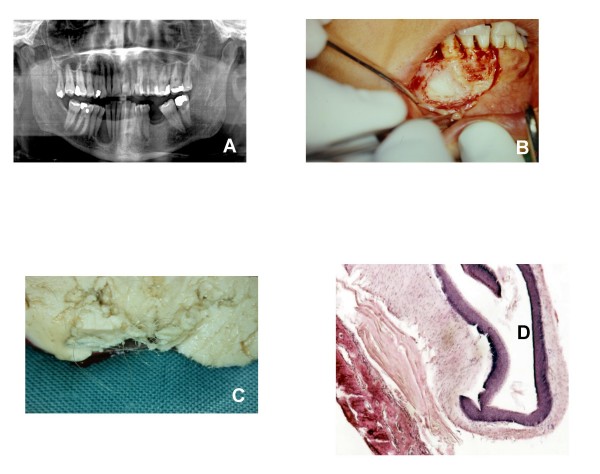
**Dermoid cyst of the mandibula: clinical, instrumental and morphological aspects.** A) Radiograph showing a radiotrasparent, monolocular area on the right side of the mandibula, located between the roots of a canine and the first premolar. B) After intra-oral incision, a "bone window" was peformed, that allowed the vision of a round solid lesion. C) Macroscopic aspect of the removed lesion. D) Histological image of the cyst (Haematoxylin/Eosin, original magnification ×20).

A 30-year-old women was referred at the Oral Surgery Department of the Dental School of the Second University of Naples in January 2008, for the treatment of a lesion, that was incidentally seen on a routine panoramic radiograph obtained for dental care. The radiograph showed a radiotrasparent, monolocular area on the right side of the mandibula, located between the roots of a canine and the first premolar with a very large displacement of the roots of these teeth (Figure 1A). Clinically it was slightly knobbly, there was neither mobility nor necrosis of the teeth included in the area of the neoplasm; the overlying mucosa appeared normoemich, normotrophic, not bloody, and the area was not much compressible by pressure and painless on palpation. Regional lymph nodes were not involved. Surgical treatment of the lesion was chosen for the treatment of this lesion. After intra-oral incision, a "bone window" was made, that permitted the vision of a round solid lesion (Figure 1B). The lesion was removed easily by a surgical courette and without resistance to the traction. Subsequently a deep local courettage and a 2-0 silk suture were performed. The surgical specimen measured 3 × 2.5 × 2.5 cm; the outer surface was regular and light-brown in colour. The cut surface showed the cystic lumen containing a keratin-like yellow (cheese-like) material (Figure 1C). The excised biopsy tumour specimen was fixed in 10% buffered-formalin and paraffin embedded. Sections of 5 μm were stained with haematoxylin-eosin, haematoxylin-van Gieson, and PAS-haematoxylin. Microscopic examination showed a cystic structure; the cyst walls were lined with a layer of keratinized squamous epithelium with the inner surface lined with keratin lamellas and the outer surface lined with gingival connective tissue components; fragments of bone tissues from the eroded mandibula were also evident (Figure 1D). The histological picture described was suggestive of a epidermoid cyst of the mandibula. The patient was discharged on post-operative day 2 with no complication reported. The surgical wound healed in 2 weeks with normal scarring.

## Discussion

The occurrence of dermoid cysts in the oral cavity is extremely rare [1-5]. Differential diagnosis should include other processes with similar characteristics and situation, such as developmental, neoplastic and infectious diseases. Histological analysis of the excised lesion is always resolutive. In most cases, dermoid cysts are treated by enucleation [6,7]. Surgical access depends on the location and size of the lesion. In the case presented, the lesion was easily enucleated after intra-oral incision through a "bone window" created by the erosion of the bone of the mandibula. To note, the intra-oral approach leads to good cosmetic and functional results; indeed, the extra-oral approach is mandatory only when the cyst lies under the geniohyoid muscle [6,7]. Prognosis is good if the cyst has been entirely eliminated; otherwise local recurrence can be observed. Malignant transformation of the dermoid cysts has been reported in some locations, but it has been never described in the mouth [1,2].

In conclusion, we describe a very rare case of dermoid cyst of the mandibula, successfully diagnosed and managed by surgical excision through intraoral access.

## Competing interests

The authors declare that they have no competing interests.

## Authors' contributions

DM and LL performed the surgical procedures and contributed to the analysis of the clinical data. NF performed the histological examination of the lesion. AB performed the histological examination of the lesion and was a major contributor in writing the manuscript together with DM. All authors read and approved the final manuscript.

## Consent

Written informed consent was obtained from the patient for publication of this case report and accompanying images. A copy of the written consent is available for review by the Editor-in-Chief of this journal.
